# Glanzmann thrombasthenia in Pakistan: molecular analysis and identification of novel mutations

**DOI:** 10.1111/cge.12622

**Published:** 2015-07-15

**Authors:** A. Haghighi, M. Borhany, A. Ghazi, N. Edwards, A. Tabaksert, A. Haghighi, N. Fatima, T.S. Shamsi, J.A. Sayer

**Affiliations:** ^1^ Department of GeneticsHarvard Medical SchoolBostonMAUSA; ^2^ Department of Medicine and the Howard Hughes Medical InstituteBrigham and Women's HospitalBostonMAUSA; ^3^ Department of HematologyHemostasis & Thrombosis of National Institute of Blood Disease & Bone Marrow TransplantationKarachiPakistan; ^4^ Chronic Pain ClinicWilderman Medicine Professional CorporationTorontoCanada; ^5^ Institute of Genetic MedicineNewcastle UniversityNewcastleUK; ^6^ Toronto General HospitalUniversity of TorontoTorontoCanada

**Keywords:** bleeding disorder, consanguineous, integrin, ITGA2B, ITGB3, Pakistan

## Abstract

Glanzmann thrombasthenia (GT) is an inherited genetic disorder affecting platelets, which is characterized by spontaneous mucocutaneous bleeding and abnormally prolonged bleeding in response to injury or trauma. The underlying defect is failure of platelet aggregation due to qualitative and/or quantitative deficiency of platelet integrin αIIbβ3 resulting from molecular genetic defects in either ITGA2B or ITGB3. Here, we examine a Pakistani cohort of 15 patients with clinical symptoms of GT who underwent laboratory and molecular genetic analysis. In patients with a broad range of disease severity and age of presentation, we identified pathogenic mutations in ITGA2B in 11 patients from 8 different families, including 2 novel homozygous mutations and 1 novel heterozygous mutation. Mutations in ITGB3 were identified in 4 patients from 3 families, two of which were novel homozygous truncating mutations. A molecular genetic diagnosis was established in 11 families with GT, including 5 novel mutations extending the spectrum of mutations in this disease within a region of the world where little is known about the incidence of GT. Mutational analysis is a key component of a complete diagnosis of GT and allows appropriate management and screening of other family members to be performed.

Glanzmann thrombasthenia (GT) is a rare mucocutaneous bleeding disorder with an autosomal recessive inheritance, first identified by Eduard Glanzmann in 1918 1. Symptoms of spontaneous bleeding vary in frequency and intensity among GT patients 2. Frequently, petechiae, purpura and easy bruising are observable features at birth 3, allowing an early clinical diagnosis. Recurrent epistaxis, gingival bleeding and menorrhagia (in females of child bearing age) are common symptoms while gastrointestinal bleeding, intracranial hemorrhage and hematuria have a lower incidental rate 2. In patients with GT, there is an increased bleeding risk after surgery and even dental procedures. Increased bleeding following accidents and trauma may also create major problems 4. Typically, the platelet count is normal 5 and platelet size remains normal in most patients, although cases with moderate thrombocytopenia and enlarged platelets have been reported 6. The combination of mucocutaneous bleeding, an absence of platelet aggregation and abnormal clot retraction associated with a normal platelet count and morphology, is diagnostic for GT 7.

Mutations in *ITGA2B* or *ITGB3* may lead to GT type I or type II, where the expression of integrin αIIbβ3 at the platelet surface is reduced (respectively less than 5% and 20% of normal). In contrast, in variant form of GT there is a functional defect of the integrin αIIbβ3 complex 8. Both *ITGA2B* and *ITGB3* are located on chromosome 17 and encode the platelet integrin αIIbβ3_,_ previously known as glycoprotein IIb/IIIa. Platelet aggregation is mediated by integrin αIIbβ3, an essential receptor for platelets. Normally, during platelet activation, the integrin αIIbβ3_,_ binds fibrinogen, which leads to platelet aggregation 9. In GT, the process of thrombus formation fails and clot retraction is also affected 9.

In patients suspected of GT, a deficiency of integrin αIIbβ3 can be confirmed using flow cytometry or western blotting with monoclonal antibodies that recognize either the αIIb, or β3 subunits or the αIIbβ3 complex 2. Genetic analysis allows a definitive confirmation of diagnosis. Both *ITGA2B* and *ITGB3* are polymorphic and are susceptible to germline mutations which occur more frequently in *ITGA2B*.

GT is a rare disease, occurring at a ratio of approximately 1:1,000,000, however it is more prevalent in certain ethnic groups 10. In this study, we analyze a cohort of 15 patients from Pakistan (from 11 different families) with GT in whom we have made a molecular genetic diagnosis. We found three novel mutations in *ITGA2B* and two novel mutations in *ITGB3* extending the molecular diagnosis in this previously uncharacterized population.

## Materials and methods

### Patients and families

A total of 15 subjects with GT from 11 different families were investigated in the study. Informed consent was obtained from all patients and relatives according to a protocol approved by the National Institute of Blood Disease & Bone Marrow Transplantation, Karachi, Pakistan. Medical histories of patients were recorded in a questionnaire, which included age, gender, age at onset, consanguineous marriage between parents, a family history of bleeding, severity of bleeding and hemorrhagic clinical symptoms.

### Hematological and molecular analysis

GT patients were screened based on platelet counts and morphology. Platelet aggregation tests were performed on a Helena AggRAM (Helena Laboratories, Beaumont, Texas, USA) using ristocetin, epinephrine, adenosine diphosphate (ADP) and collagen. Bleeding time (BT) measurements were performed and compared with reference values: 0–4 years, 4 ± 1 min; boys >4 years, 5 ± 1 min; girls >4 years, 5.5 ± 1 min. Genomic DNA was extracted from peripheral blood leukocytes isolated from whole blood obtained from patients and (where available) parents. Exon polymerase chain reaction (PCR) was used to amplify all coding regions of the *ITGA2B* and *ITGB3* genes (using reference sequences NM_000419 and NM_000212, respectively). PCR products were purified and Sanger sequenced. Segregation using parental samples was undertaken when available. MutationTaster^™^ (http://www.mutationtaster.org/) and PolyPhen‐2 (http://genetics.bwh.harvard.edu/pph2/index.shtml) were used to determine pathogenicity of novel mutations. The crystal structure of the complete ectodomain of integrin αIIbβ3 (PDB code 3FCS) 11, 12 was visualized using PyMOL (http://pymol.org/) to identify the position of the missense mutations identified in the present study.

## Results

### Clinical and laboratory findings

A total of 15 GT patients from 11 consanguineous Pakistan families were evaluated in this study (Table 1). The age of the patients ranged from 1 to 16 years with an age of disease onset ranging from 7 days to 4 years (mean age of onset of 2 years). Fifty percentage of patients had a positive family history of a bleeding disorder. Common clinical symptoms included recurrent epistaxis and gingival bleeding (73%), and easy bruising (67%) (Table 1). Gastrointestinal hemorrhage (four cases), petechiae and purpura (two cases) and hemathrosis (one case) were rare. BT was prolonged in all of our patients and was often over 10 min, while activated partial thromboplastin time (APTT) and prothrombin time (PT) remained within normal range in 14 (93%) and 13 (87%) patients, respectively. Platelet aggregation assays for all cases measured were defective when using ADP, adrenaline, collagen and arachidonic acid. These findings are typical of GT, as platelet aggregation tends to be defective with all agonists. The response to ristocetin was maintained in 13 of 14 patients tested and platelet count was within normal limits for all patients except one (Table 1).

**Table 1:** Clinical and laboratory findings in the GT cohort

Patient ID	A	B	C	D	E	F	G	H	I	J	K	L	M	N	O
Family number	1	2	3	3	4	5	6	7	8	8	8	9	9	10	11
Age (years), sex	6, M	16, M	12, M	10, M	1, F	16, F	1, F	8, F	3, F	5, F	5, M	10, F	15, F	7, M	7, F
Age at onset	4.5 y	5 d	2 y	1 y	3 m	12 y	6 m	6 m	3 m	6 m	7 d	4 y	4 y	1 y	1 y
Clinical symptoms															
Petechiae and purpura	−	−	−	−	−	−	−	−	+	−	−	−	−	+	−
Easy bruising	+	−	−	−	+	+	+	+	+	+	+	+	+	+	−
Recurrent epistaxis	−	+	−	+	−	+	+	+	+	+	+	−	+	+	+
Gingival bleeding	−	+	−	−	+	+	+	+	−	+	+	+	+	+	+
Gastrointestinal bleeding	+	−	−	−	−	−	−	+	−	+	+	−	−	−	−
Intracranial hemorrhage	−	−	−	−	−	−	−	−	−	−	−	−	−	−	−
Hematuria	−	−	−	−	−	−	−	−	−	−	+	−	−	−	−
Hemorrhage after dental	−	−	−	−	−	−	−	+	−	−	−	−	−	+	−
Hemorrhage after trauma	−	−	−	−	−	−	−	+	−	+	+	−	−	+	−
Ear bleeding	−	−	−	−	−	−	+	+	−	−	+	−	−	−	−
Hemarthrosis	−	−	−	−	−	−	−	+	−	−	−	−	−	−	−
Hematemesis	−	−	−	−	−	−	−	−	−	−	−	−	−	−	−
Hematoma	−	−	+	−	−	−	−	−	−	+	+	−	−	−	−
Bleeding time (min) (for NR see Methods)	10	7	10	N/A	>10	N/A	>10	>10	>10	10	10	10	>10	>10	N/A
APTT (s) (NR 27–35)	29	28	28	26	28	**38**	29	29	30	28	29	29	25	25	30
PT (s) (NR 12–13)	12.5	**14**	12	11.5	12	**16**	12.5	11.5	11.5	13	11	12	13	12	12
Platelet aggregation assay															
ADP	X	X	X	N/A	X	X	X	X	X	X	X	X	X	X	X
Adrenaline	X	X	X	N/A	X	X	X	X	X	X	X	X	X	X	X
Collagen	X	X	X	N/A	X	X	X	X	X	X	X	X	X	X	X
Arachidonic acid	X	X	N/A	N/A	X	X	X	X	X	X	X	X	X	X	X
Ristocetin	N	N	N	N/A	N	N	X	N	N	N	N	N	N	N	N
Hematological evaluation															
Platelet count (× 10^9^/l) (NR 150–450)	371	205	156	150	212	235	407	312	212	**129**	272	225	199	250	301
Red blood cell count (× 10^6^/ml)	3.9	3.7	3.4	3.7	3.2	2.5	4.12	3.93	3.69	3.1	3.57	3.2	3.5	2.6	3.8
Family history of bleeding	−	+	−	N/A	−	−	+	−	+	+	+	+	+	−	−

+, present; −, absent; ADP, adenosine diphosphate; d, day; F, female; GT, Glanzmann thrombasthenia; m, month; M, male; min, minutes; N, normal; N/A, not available; NR, Normal range; s, second; X, defective; y, year. Abnormal results are shown in bold.

### Genetic findings

**Figure 1:**
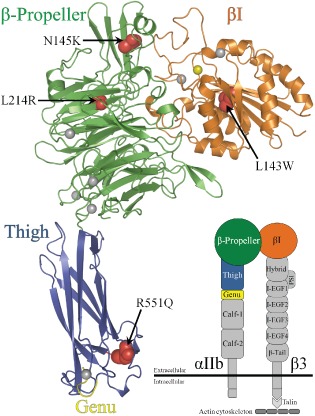
Molecular modeling of missense mutations affecting the integrin αIIbβ3 complex. Crystal structure of the αIIbβ3 ectodomain [PDB:3FCS]. The domains are color‐coded to match the schematic (inset) showing the complete integrin structure. Ca^2+^ and Mg^2+^ ions are represented as yellow and gray spheres, respectively. The mutations identified in this study are highlighted as red spheres.

We identified 7 different mutations in *ITGA2B* in 11 patients (from 8 families). Three of these mutations in this gene (p.N145K, p.S477Gfs*111 and p.N536Kfs*29) were novel (Table 2) and were not found on database searches including 1000 Genomes (http://www.1000genomes.org/), Exome Variant Server (http://evs.gs.washington.edu/EVS/) and the GT database (http://sinaicentral.mssm.edu/intranet/research/glanzmann/menu). One patient (A, Family 1) had compound heterozygous mutations which included the novel missense change p.N145K together with a nonsense mutation, p.C177* previously reported by Jallu et al. (in its homozygous state) in a GT patient from China 13. Unfortunately, parental samples were unavailable to confirm segregation, but given the negative family history of GT in this family, it is possible that one of these mutations may have occurred *de novo*. The remaining patients had homozygous mutations, in keeping with known consanguinity. Mutations included p.L214R, which has been previously reported and investigated in terms of its pathogenicity 14. The nonsense mutation p.Y411* found in two siblings of Family 3 and is predicted to lead to a severe truncation of the αIIb protein. This mutation has previously been reported in a heterozygous state (in combination with a p.V329F missense mutation) in a 1‐year‐old child with GT 15. Both p.S477Gfs*111 (Family 4) and p.N536Kfs*29 (Family 5), secondary to a small insertions/deletions, respectively, were novel mutations and were predicted to disrupt the extracellular thigh domain of integrin αIIb (Fig. 1, Table 2). The previously reported R551Q missense mutation which caused severe disease at birth in an Indian child 16 was present in five patients from three different families (Fig. 1, Table 2). The presentation of these cases was between 7 days of age and 6 months of age.

**Table 2:** Molecular genetic findings in the GT cohort

Patient ID	A	B	C	D	E	F	G	H	I	J	K	L	M	N	O
Family number	1	2	3	3	4	5	6	7	8	8	8	9	9	10	11
Gene	ITGA2B	ITGA2B	ITGA2B	ITGA2B	ITGA2B	ITGA2B	ITGA2B	ITGA2B	ITGA2B	ITGA2B	ITGA2B	ITGB3	ITGB3	ITGB3	ITGB3
Homozygous/Heterozygous	Compound Het	Homo	Homo	Homo	Homo	Homo	Homo	Homo	Homo	Homo	Homo	Homo	Homo	Homo	Homo
Nucleotide change^a^	**c.435C>A**/c.531 T > A	c.641 T>G	c.1265C>T	c.1265C>T	**c.1460insAGGT**	**c.1608delT**	c.1684G>A	c.1684G>A	c.1684G>A	c.1684G>A	c.1684G>A	c.448 T>G	c.448 T>G	**c.793 T>A**	**c.2080C>T**
Amino acid change^a^	**p.N145K**/p.C177*	p.L214R	p.Y411*	p.Y411*	p.S477Gfs*111	p.N536Kfs*29	p.R551Q	p.R551Q	p.R551Q	p.R551Q	p.R551Q	p.L143W	p.L143W	p.C258*	p.Q694*
Parental consanguinity	+	+	+	+	+	+	+	+	+	+	+	+	+	+	+
Segregation	N/A	N/A	+	+	+	N/A	+	+	+	+	+	+	+	+	N/A
Novel/reference	**Novel/**Jallu et al. 13	Grimaldi et al. 14	Mitchell et al. 15 (annotated as Y380*)	Mitchell et al. 15 (annotated as Y380*)	**Novel**	**Novel**	Vijapurkar et al. 16	Vijapurkar et al. 16	Vijapurkar et al. 16	Vijapurkar et al. 16	Vijapurkar et al. 16	Basani et al.^17^	Basani et al. 17	**Novel**	**Novel**
MutationTaster (score)^a^	**Disease causing (0.999)**	Disease causing (1.000)	Disease causing (1.000)	Disease causing (1.000)	**Disease causing (1.000)**	**Disease causing (1.000)**	Disease causing (0.999)	Disease causing (0.999)	Disease causing (0.999)	Disease causing (0.999)	Disease causing (0.999)	Disease causing (0.999)	Disease causing (0.999)	**Disease causing** **(1.000)**	**Disease causing** **(1.000)**
Polyphen2 (score)^a^	Probably damaging **(1.000)**	Probably damaging (1.000)	N/A	N/A	N/A	N/A	Probably damaging (1.000)	Probably damaging (1.000)	Probably damaging (1.000)	Probably damaging (1.000)	Probably damaging (1.000)	Probably damaging (1.000)	Probably damaging (1.000)	N/A	N/A
Location of mutation within protein domains	Within beta propeller repeat	Within beta propeller repeat	Within beta propeller repeat	Within beta propeller repeat	Within Thigh domain	Within Thigh domain	Within Thigh domain	Within Thigh domain	Within Thigh domain	Within Thigh domain	Within Thigh domain	Within integrin beta subunit	Within integrin beta subunit	Within integrin beta subunit	Within integrin beta subunit

GT, Glanzmann thrombasthenia; N/A, not available.

a Novel nucleotide changes, amino acid changes, and scores using MutationTaster and PolyPhen2 are shown in bold.

We identified three different mutations in *ITGB3* in four patients (from three families) which are predicted to affect the integrin beta subunit (Fig. 1, Table 2). All mutations in *ITGB3* were homozygous and two mutations were novel nonsense mutations (p.C258* and p.Q694*) not found in database searches. The L143W mutation in *ITGB3* was found in two patients, and has been previously described in a patient also from Pakistan 17 and in two patients from India 18.

## Discussion

GT is an autosomal recessively inherited platelet function disorder that appears to be more common in areas with high consanguinity rate. Consanguineous unions are commonly seen among Pakistanis, as in other ethnic groups including Iraqi Jews, Northern Iranians, Southern Indians, Jordanians, Saudi Arabians and Arabs living in Israel. The Pakistan Demographic and Health Survey revealed that two thirds of marriages in Pakistan are consanguineous, promoting increased rates of rare autosomal recessive disorders 19. In our study, all of the cases were born to consanguineous parents.

Most of the GT cases are diagnosed clinically at an early age. Our study had an age of presentation from 7 days to 4 years and half of the patients had a history of severe bleeding. All our patients with GT showed typical defective platelet aggregometry in response to the agonists used. A differential diagnosis, especially in older patients, would include the development of inhibitory anti‐αIIbβ3 autoantibodies that cause an acquired thrombasthenia 20. Leukemia may also present in a similar manner in later life and investigations of platelet function should be performed in a systematic fashion. Finally, there are reports of an autosomal dominant GT‐like syndrome which displays macrothrombocytopenia 21. Platelet counts were decreased in one of our patients and two patients had prolonged PT. Some molecular defects in GT may affect platelet production, such as the R560B3 defect that was recently modeled *in vivo* 22.

We identified a wide spectrum of mutations within *ITGA2B* and *ITGB3*. Several families identified here had novel ‘private’ mutations, emphasizing a need to sequence the entire coding regions of both genes. The establishment of infrastructures to allow such molecular genetic diagnostics in remote regions of the world, where consanguinity rates are high, would be a real advance.

Integrins have two subunits, alpha and beta and form an obligate heterodimer (Fig. 1). In mammals, there are 18 different alpha and 8 different beta subunits. Integrin subunits penetrate the plasma membrane and have short (40–70 amino acid) cytoplasmic domains 23. The integrin αIIbβ3 complex is formed from a calcium‐dependent association of glycoprotein IIb and IIIb. The crystal structure of integrin αIIbβ3 has been described 11, 12. Mapping of known mutations to the functional domains of the integrin αIIbβ3 complex shows that a broad range of mutations exists and that genotype/phenotype correlations are difficult within this complex molecular structure.

## Conclusion

GT occurs in high frequency in certain ethnic populations with an increased incidence of consanguinity. GT is caused by mutations in the genes encoding *ITGA2B* or *ITGB3* that result in qualitative or quantitative abnormalities in the platelet membrane proteins leading to bleeding disorders. A precise molecular diagnosis in patients with suspected GT allows an increased understanding of the disease pathogenesis, and is important for screening of at risk family members and establishing a prenatal diagnosis. Here, we have successfully identified pathogenic mutations in either *ITGA2B* or *ITGB3* in 15 patients with GT from a previously uncharacterized ethnic group and country, where little is known about the incidence and molecular genetics of GT.
